# SUMOylation Protects Endothelial Cell-Expressed Leukocyte-Specific Protein 1 from Ubiquitination-Mediated Proteasomal Degradation and Facilitates Its Nuclear Export

**DOI:** 10.3390/ijms27021111

**Published:** 2026-01-22

**Authors:** Mokarram Hossain, Jiannan Huang, Yang Su, Md Rafikul Islam, Mohammad Alinoor Rahman, Francisco S. Cayabyab, Lixin Liu

**Affiliations:** 1 Department of Anatomy, Physiology and Pharmacology, College of Medicine, University of Saskatchewan, Saskatoon, SK S7N 5E5, Canada; jih581@mail.usask.ca (J.H.); suyang0922@hotmail.com (Y.S.); lixin.liu@usask.ca (L.L.); 2 Department of Biochemistry and Molecular Biology, College of Medicine, University of Arkansas for Medical Sciences, Little Rock, AR 72205, USA; mrislam@uams.edu (M.R.I.); marahman@uams.edu (M.A.R.); 3 Department of Surgery, College of Medicine, University of Saskatchewan, Saskatoon, SK S7N 5E5, Canada; frank.cayabyab@usask.ca

**Keywords:** leukocyte-specific protein 1, SUMOylation, posttranslational modification, SUMO1, endothelial permeability

## Abstract

Leukocyte-specific protein 1 (LSP1) is known as an endothelial gatekeeper because it controls endothelial permeability and transendothelial cell migration, including that of leukocytes and potentially metastatic cancer cells. In endothelial cells, LSP1 is predominantly in the nucleus under resting conditions but translocates to extranuclear compartments upon stimulation with TNF-α. The discrepancy between its predicted molecular weight (~37 kDa) and its observed migration on SDS-PAGE (≥52 kDa), along with its dynamic subcellular distribution, suggests a possible post-translational modification by SUMOylation. To investigate this, we examined endogenous LSP1 in murine primary endothelial cells and overexpressed recombinant LSP1 in murine endothelial (SVEC4-10EE2) and HEK293T cells. Our results demonstrate that LSP1 is SUMOylated by SUMO1, with Ubc9 serving as the conjugating enzyme and SENP1 as the deSUMOylating protease. Site-directed mutagenesis of lysines K270 and K318 abolished SUMOylation, resulting in a marked reduction in LSP1 steady-state levels. This reduction was attributed to enhanced ubiquitination and accelerated proteasomal degradation of LSP1 in the SUMOylation-deficient state. Furthermore, deSUMOylation impaired the TNF-α-induced translocation of LSP1 from the nucleus to extranuclear compartments, particularly the cytoskeleton. In summary, our findings establish that LSP1 is a SUMO1-modified protein. SUMOylation stabilizes LSP1 by preventing proteasomal degradation and is essential for its proper subcellular trafficking in endothelial cells in response to inflammatory stimuli.

## 1. Introduction

Leukocyte-specific protein 1 (LSP1) was initially identified in B and T lymphocytes [1,2]. Later, LSP1 was found to be expressed in a variety of immune and other cells, including thymocytes, monocytes, macrophages, dendritic cells, neutrophils, and endothelial cells [3,4,5,6]. It is an intracellular protein that binds both Ca^2+^ and filamentous actin (F-actin). Human and mouse LSP1 proteins consist of 339 and 330 amino acids, respectively, and share approximately 67% sequence identity [2,7,8]. While the N-terminal region, which contains Ca^2+^-binding sites, shows moderate conservation between species (53% identity), the C-terminal region, rich in basic amino acids and harboring two F-actin–binding domains, is highly conserved (85% identity) [1,9]. In endothelial cells, it is predominantly a nuclear protein with a minor fraction associated with F-actin-rich cytoskeletal microfilaments [6]. Upon TNF-α stimulation, endothelial LSP1 translocates from the nucleus to extranuclear compartments, particularly the cytoskeleton [10].

Functionally, LSP1 plays a key role in regulating leukocyte migration, endothelial permeability, and tumor progression [6,11,12,13]. LSP1 reorganizes existing F-actin into bundles, facilitating directional movement of cells [14,15]. Given its dual role in controlling endothelial permeability and leukocyte trafficking, LSP1 is increasingly implicated in cancer metastasis, a process in which tumor cells exploit mechanisms similar to those of directional leukocyte migration and transendothelial migration. Recent studies have begun to explore LSP1’s involvement in cancer [12,13], with particular interest in its potential role in metastasis, though this remains an emerging area of research.

Small ubiquitin-like modifiers (SUMO) are a family of proteins that regulate target protein function by covalently binding to specific lysine residues [16]. In vertebrates, the SUMO family includes SUMO1, SUMO2, SUMO3, and SUMO4 [17]. SUMOylation commonly occurs at a conserved ΨKxD/E motif, although non-canonical motifs can also be modified [18,19]. The SUMOylation process involves a cascade of enzymes: activation by E1 enzymes (SAE1/SAE2), conjugation by the sole E2 enzyme Ubc9, and ligation by E3 ligases (e.g., PIAS proteins), which enhance substrate specificity and efficiency. SUMOylation is reversible, with SUMO-specific proteases (SENPs) responsible for cleaving SUMO from substrates [16,19,20,21]. This dynamic modification regulates a wide range of cellular processes, including transcription, DNA repair, signal transduction, protein stability, localization, and trafficking [16,22,23,24,25,26,27,28].

Although murine LSP1 is predicted to have a molecular weight of 37 kDa, it appears at a higher molecular weight (≥52 kDa) in SDS-PAGE. This discrepancy suggests the presence of posttranslational modifications, which are known to influence both protein molecular weight [16,29] and subcellular localization [20,30,31]. The marked shift in molecular weight (15 kDa), combined with LSP1’s translocation from the nucleus to extranuclear compartments following TNF-α treatment [10], raises the possibility that LSP1 is posttranslationally modified by SUMOylation.

To address this, we investigated whether LSP1 is SUMOylated in both naive endothelial cells and in overexpression systems. Using bioinformatic tools, we identified candidate SUMOylation sites on LSP1 and validated these findings through site-directed mutagenesis. We further examined the role of SUMOylation in regulating LSP1 stability and subcellular localization, specifically nuclear export, in endothelial cells.

## 2. Results

### 2.1. LSP1 Is SUMOylated

**Figure 1:**
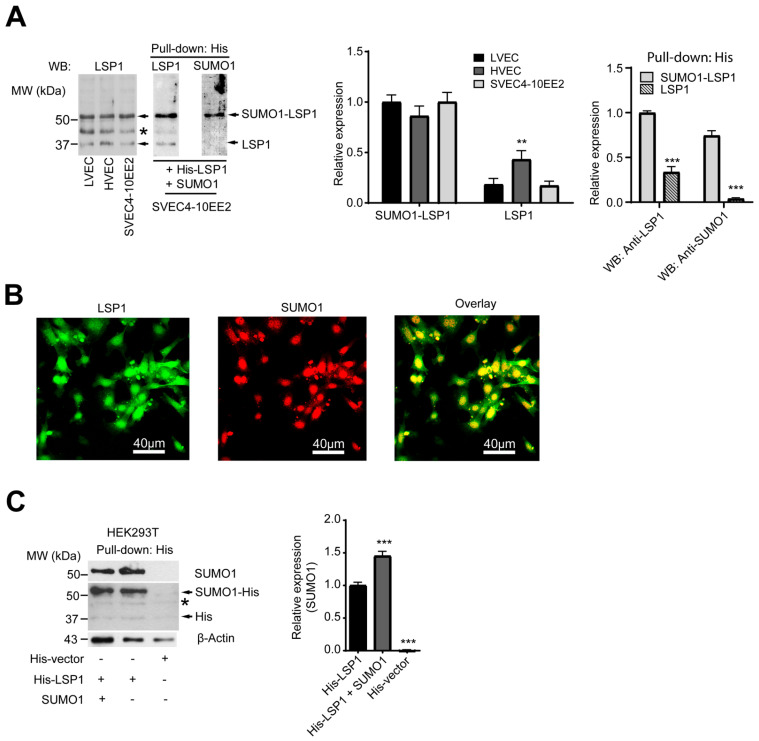
LSP1 is modified by SUMO1. (**A**) Representative (*n* = 3) original Western blots showing LSP1 protein expression in murine primary vascular endothelial cells isolated from heart (HVEC) and lungs (LVEC) of 129/SvJ mice and untransfected murine endothelial cell line (SVEC4-10EE2) cells (left); LSP1 (middle) and SUMO1 (right) protein expression in pulled-down His-tagged proteins from His-LSP1- and SUMO1-overexpressed SVEC4-10EE2 cells. The arrow indicates LSP1 or SUMO1-LSP1 conjugate, and * indicates an unknown signal. Blots were analyzed by densitometry; results (mean ± SD; *n* = 3) of three independent experiments were plotted as relative expression (right). ** indicates *p* < 0.01 and *** indicates *p* < 0.001. (**B**) Representative (*n* = 3) confocal images of LSP1 (Green; left), SUMO1 (Red; middle), and overlay (right) in LSP1- and SUMO1-overexpressed SVEC4-10EE2 cells. (**C**) Representative (*n* = 3) original Western blot showing SUMO1 (top) and His (middle) protein expression in pulled-down His-tagged proteins and β-actin (bottom) protein expression in the whole cell lysate from His-LSP1- or His-LSP1 and SUMO1-overexpressed HEK293T cells. The arrow indicates His or SUMO1-His conjugate, and * indicates an unknown signal. Blots were analyzed by densitometry; results (mean ± SD; *n* = 3) of three independent experiments were plotted as relative expression (right). *** indicates *p* < 0.001.

Three distinct bands at approximately 37 kDa, 45 kDa, and 52 kDa were detected in whole cell lysates of mouse primary endothelial cells isolated from lungs and hearts when immunoblotted with anti-LSP1 rabbit serum (a gift from Dr. Jongstra, University of Toronto). These bands were similarly observed in both naïve and LSP1-overexpressing SVEC4-10EE2 endothelial cells (Figure 1A), suggesting either post-translational modifications of LSP1 or potential non-specific binding of the anti-LSP1 antibody. To investigate this further, the corresponding bands were excised from a Coomassie Blue–stained gel and analyzed by mass spectrometry. The analysis confirmed the presence of LSP1 in both the 37 kDa band (Mowse/ion score: 89; matched queries: 2; sequence coverage: 7%; pI/molecular weight: 4.7/36.7) and the 52 kDa band (Mowse/ion score: 98; matched queries: 3; sequence coverage: 11%; pI/molecular weight: 4.7/36.7). The ion score, calculated as −10 × log(P), where P is the probability that the observed match is a random event, indicates that a score greater than 32 reflects identity or extensive homology (*p* < 0.05). However, mass spectrometric analysis of the 45 kDa band was unsuccessful.

Immunoblot analysis of His-tagged proteins pulled down from lysates of SVEC4-10EE2 cells co-overexpressing His-LSP1 and SUMO1, using a monoclonal anti-SUMO1 antibody, revealed a single band at approximately 52 kDa, consistent with SUMOylated LSP1 (Figure 1A). Furthermore, confocal microscopy confirmed the co-localization of GFP-LSP1 and SUMO1 in SVEC4-10EE2 cells co-transfected with GFP-LSP1 and SUMO1 (Figure 1B). Similar results were obtained in HEK293T cells overexpressing His-LSP1 and SUMO1 (Figure 1C). Taken together, these findings indicate that LSP1 undergoes SUMO1-mediated modification in endothelial cells.

### 2.2. LSP1 SUMOylation Is Facilitated by Ubc9, Whereas Its deSUMOylation Is Mediated by SENP1

**Figure 2:**
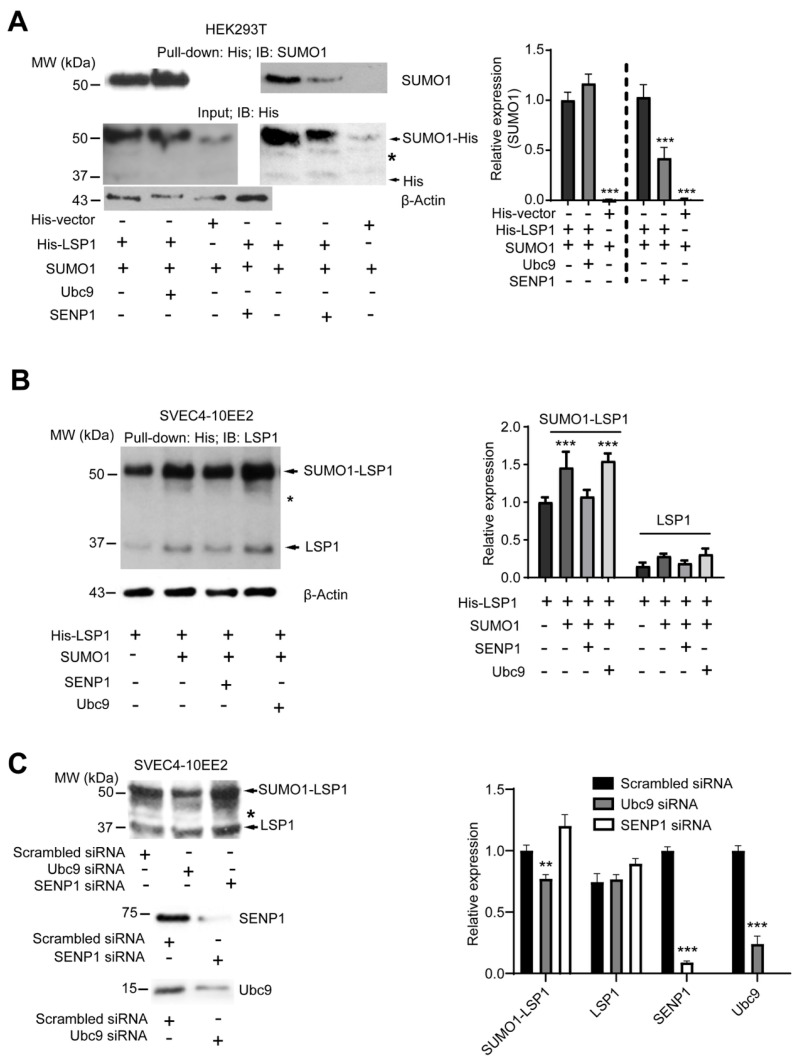
Ubc9 acts as a SUMO conjugase, and SENP1 acts as a SUMO protease for LSP1. (**A**) Pulled-down His-tagged proteins from His-LSP1 and SUMO1-; His-LSP1, SUMO1, and Ubc9- or His-LSP1, SUMO1, and SENP1-overexpressed HEK293T cells were immunoblotted with a monoclonal anti-SUMO1 or a polyclonal anti-His antibody. β-actin in the whole-cell lysate was detected to ensure equal amounts of total protein were used for pulling down His-tagged proteins. Co-expression of Ubc9 (left) and SENP1 (right) substantially increased and decreased the LSP1-SUMO-1 conjugation, respectively (*n* = 3). The arrow indicates His or SUMO1-His conjugate, and * indicates an unknown signal. Blots were analyzed by densitometry; results (mean ± SD; *n* = 3) of three independent experiments were plotted as relative expression (right). *** indicates *p* < 0.001. (**B**) Pulled-down His-tagged proteins from His-LSP1 and SUMO1-; His-LSP1, SUMO1 and Ubc9-; His-LSP1, SUMO1 and SENP1- or His-LSP1-overexpressed SVEC4-10EE2 cells were immunoblotted with anti-LSP1 rabbit serum. β-actin in the whole-cell lysate was detected to ensure equal amounts of total protein were used for pulling down His-tagged proteins. Co-expression of SUMO1 and SENP1 significantly increased and reduced the LSP1-SUMO1 conjugation, respectively (*n* = 3). The arrow indicates LSP1 or SUMO1-LSP1 conjugate, and * indicates an unknown signal. Blots were analyzed by densitometry; results (mean ± SD; *n* = 3) of three independent experiments were plotted as relative expression (right). *** indicates *p* < 0.001. (**C**) Endogenous SENP1 and Ubc9 were knocked down in SVEC4-10EE2 cells using siRNA to assess their roles in LSP1 SUMOylation. Silencing of Ubc9 resulted in reduced LSP1 SUMOylation, whereas silencing of SENP1 led to increased LSP1 SUMOylation. LSP1 was detected by immunoblotting using an anti-LSP1 antibody (Santa Cruz Biotechnology). Blots were analyzed by densitometry; results (mean ± SD; *n* = 3) of three independent experiments were plotted as relative expression (right). ** indicates *p* < 0.01 and *** indicates *p* < 0.001.

Ubc9 is the sole E2 conjugating enzyme responsible for directing SUMO molecules to target proteins. To determine whether Ubc9 mediates the SUMOylation of LSP1, we co-expressed SUMO1 and His-LSP1 with or without Ubc9 in HEK293T cells. Immunoblot analysis revealed that co-transfection with Ubc9 markedly enhanced the SUMOylation of His-LSP1 (Figure 2A, left panel), indicating that Ubc9 functions as the SUMO1-conjugating enzyme for LSP1.

SUMOylation is a reversible process, and SUMO moieties are removed from target proteins by specific SUMO proteases (SENPs) in an ATP-dependent manner. SENP1 is recognized as the primary protease responsible for cleaving SUMO1 [32]. To evaluate whether SENP1 regulates LSP1 deSUMOylation, we co-expressed His-tagged LSP1 and SUMO1, with or without SENP1, in HEK293T cells. As shown in Figure 2A (right panel), expression of SENP1 significantly reduced the level of SUMOylated LSP1, suggesting that SENP1 facilitates the deSUMOylation of LSP1.

Consistent results were obtained in SVEC4-10EE2 endothelial cells, where overexpression of Ubc9 and SENP1 similarly modulated LSP1 SUMOylation (Figure 2B). Collectively, these findings provide strong evidence that Ubc9 and SENP1 function as the SUMO conjugating enzyme and deSUMOylating protease, respectively, in the regulation of LSP1 SUMOylation.

We also knocked down SENP1 and Ubc9 in SVEC4-10EE2 endothelial cells using siRNA to validate our findings from the overexpression system. The siRNA-mediated knockdown experiments mirrored the results of the overexpression studies, providing strong evidence that Ubc9 and SENP1 act as the SUMO conjugase and SUMO protease, respectively, in regulating LSP1 SUMOylation (Figure 2C).

### 2.3. K270 and K318 Are the Primary Sites of LSP1 SUMOylation

After confirming that LSP1 undergoes posttranslational modification by SUMO1, we sought to identify the specific SUMO1 acceptor sites on LSP1. To predict potential SUMOylation sites, we performed bioinformatics analysis using SUMOplot™ (http://www.abgent.com/sumoplot; accessed 30 June 2024), as previously described [22,23,33]. This analysis identified a high-probability SUMOylation site at lysine (K) 318 in mouse LSP1. In contrast, SUMOplot™ predicted two high-probability SUMOylation sites in human LSP1 at K279 and K327, corresponding to K270 and K318 in mouse LSP1, respectively—both of which are evolutionarily conserved (Table 1). Based on these findings, K270 and K318 were selected as putative SUMOylation sites in murine LSP1.

**Figure 3:**
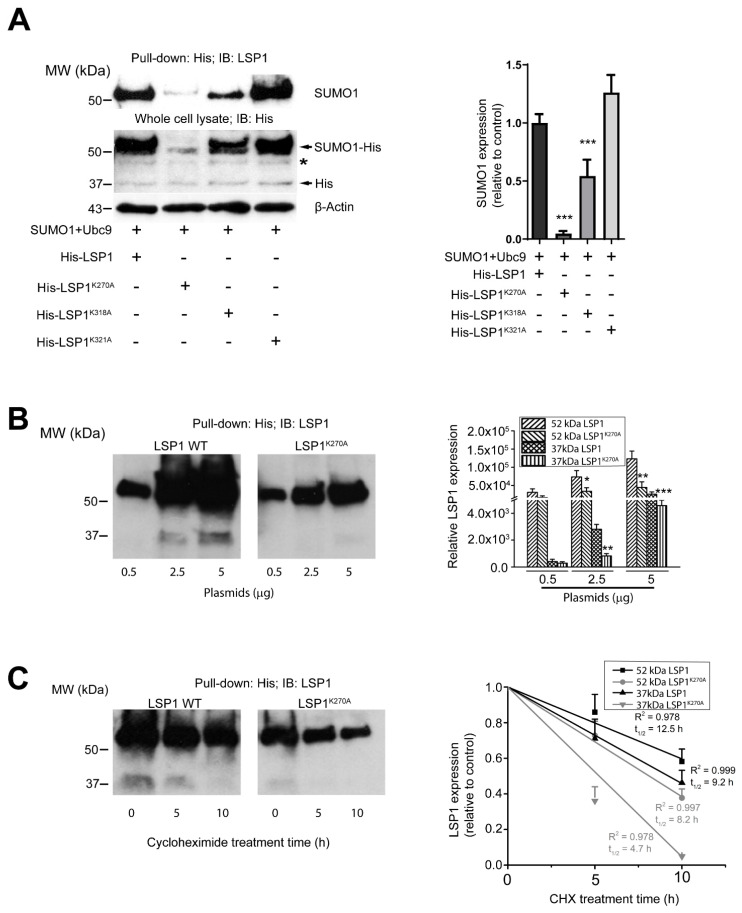
Lysine residues 270 and 318 are the primary sites of LSP1 SUMOylation. (**A**) Pulled-down His-tagged proteins from SUMO1, Ubc9, and His-LSP1- or His-LSP1^K270A^- or His-LSP1^K318A^- or His-LSP1^K321A^-co-expressed HEK293T cells were immunoblotted with a monoclonal anti-SUMO1 or a polyclonal anti-His antibody. β-actin in the whole-cell lysate was detected to ensure equal amounts of total protein were used for pulling down His-tagged proteins (*n* = 3). The arrow indicates His or SUMO1-His conjugate, and * indicates an unknown signal. Blots were analyzed by densitometry; results (mean ± SD; *n* = 3) of three independent experiments were plotted as relative expression (right). *** indicates *p* < 0.001. (**B**) HEK293T cells were transfected with a range (0.5–5 µg) of His-LSP1 or His-LSP1^K270A^ plasmid quantity. Twelve hours after transfection, cells were lysed, and proteins were immunoblotted with a polyclonal anti-LSP1 antibody (left). Blots were analyzed by densitometry; results (mean ± SD; *n* = 3) of three independent experiments were plotted as arbitrary units (right). * indicates *p* < 0.05, ** indicates *p* < 0.01 and *** indicates *p* < 0.001 from wild-type LSP1. (**C**) Twelve hours after His-LSP1 or His-LSP1^K270A^ transfection, HEK293T cells were treated with 100 mg/mL cycloheximide (CHX) for the indicated periods. Cells were lysed, and proteins were immunoblotted using an anti-LSP1 antibody (relative to β-actin; left). Blots were analyzed by densitometry; results (mean ± SD; *n* = 3) of three independent experiments were plotted as arbitrary units (relative to 0 h expression), and half-lives (t_1/2_) were calculated by linear regression analysis of the fitted curves (right).

To ensure that the introduction of mutations did not nonspecifically alter LSP1 expression, we also introduced a random mutation at K321. Site-directed mutagenesis was used to generate three His-tagged LSP1 variants: K270A (His-LSP1-K270A), K318A (His-LSP1-K318A), and K321A (His-LSP1-K321A). These constructs were co-expressed with SUMO1 and Ubc9 in HEK293T cells to evaluate the functional relevance of these lysine residues in LSP1 SUMOylation. Immunoblot analysis of His-tagged proteins pulled down with a monoclonal anti-SUMO1 antibody revealed that the mutation at K270 nearly abolished SUMOylation, the mutation at K318 significantly reduced it, while the mutation at K321 had no discernible effect (Figure 3A). These results indicate that K270 and K318 serve as the primary SUMOylation sites in murine LSP1.

### 2.4. DeSUMOylation Affects LSP1 Stability

After 24 h of transfection, the His-tagged protein band at 52 kDa was almost completely absent and significantly reduced in cells transfected with His-LSP1K270A and His-LSP1K318A compared to those transfected with wild-type His-LSP1. However, there was no corresponding increase in the 37 kDa His-tagged protein band in these cells (Figure 3A), suggesting that mutations in the SUMO1 acceptor sites of LSP1 may significantly impact the steady-state levels of recombinant LSP1.

To investigate this, we transfected HEK293T cells with equal amounts of His-LSP1 or His-LSP1K270A plasmids. Cells transfected with His-LSP1K270A showed significantly lower levels of LSP1 protein (37 kDa) in total cell lysates compared to those transfected with His-LSP1 across various plasmid concentrations (0.5, 2.5, and 5 μg/mL) (Figure 3B). This observation indicates that reduced SUMOylation results in lower steady-state levels of LSP1. Changes in steady-state protein levels can result from alterations in either protein synthesis or degradation [34]. To further explore the role of SUMOylation in LSP1 stability, we transfected HEK293T cells with wild-type His-LSP1 or His-LSP1K270A plasmids. After 12 h, the cells were treated with cycloheximide for varying durations to halt further protein synthesis. Cycloheximide is commonly used to study the degradation kinetics of specific proteins over time [16,22,23,35]. The treated cells were lysed at designated time points and analyzed by immunoblotting with an anti-LSP1 antibody. Densitometric quantification showed that mutant LSP1K270A degraded at a much faster rate than the wild-type LSP1. The half-life of mutant LSP1K270A was calculated to be just 4.7 h, compared to 9.2 h for wild-type LSP1 (Figure 3C).

**Figure 4:**
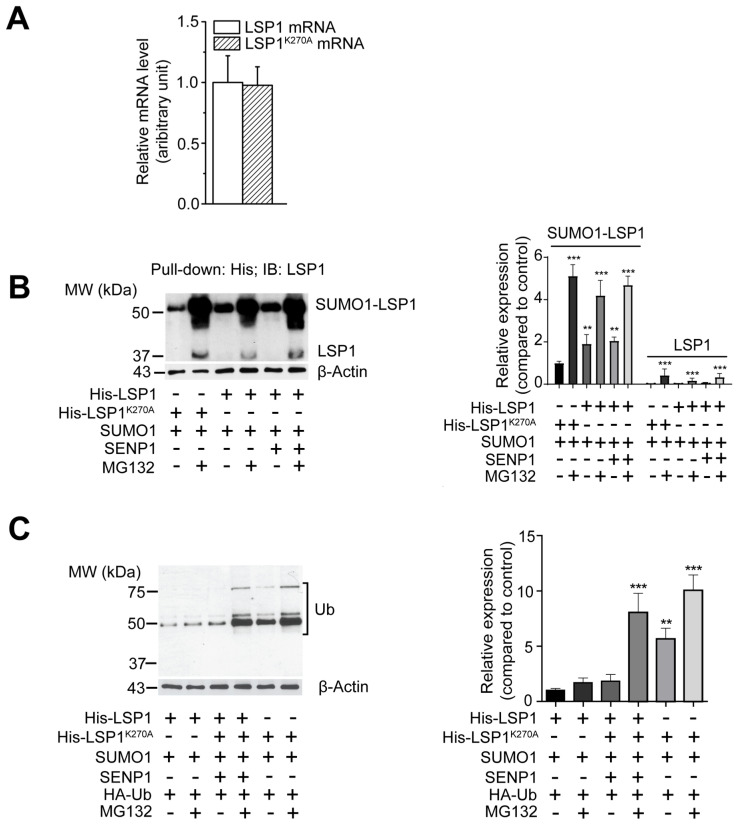
DeSUMOylation leads to polyubiquitination and subsequent proteasomal degradation of LSP1. (**A**) Mean ± SD of mRNA levels (*n* = 3) encoding LSP1 in His-LSP1- or His-LSP1^K270A^-transfected HEK293T cells. (**B**) HEK293T cells were transfected with His-LSP1 and SUMO1; His-LSP1, SUMO1, and SENP1 or His-LSP1^K270A^ and SUMO1 plasmids. After 12 h, cells were treated with or without 10 µM MG132 for 12 h, lysed, and proteins were immunoblotted with an anti-LSP1 antibody (relative to β-actin; *n* = 3). Blots were analyzed by densitometry; results (mean ± SD; *n* = 3) of three independent experiments were plotted as relative expression (right). ** indicates *p* < 0.01 and *** indicates *p* < 0.001. (**C**) HEK293T cells were transfected with His-LSP1, SUMO1, and HA-Ub; His-LSP1, SUMO1, SENP1, and HA-Ub or His-LSP1^K270A^, SUMO1, and HA-Ub plasmids. After 12 h, cells were treated with or without 10 µM MG132 for 12 h, lysed, and proteins were immunoblotted with an anti-ubiquitin antibody (relative to β-actin; *n* = 3). Blots were analyzed by densitometry; results (mean ± SD; *n* = 3) of three independent experiments were plotted as relative expression (right). ** indicates *p* < 0.01 and *** indicates *p* < 0.001.

Additionally, to determine if the mutation of the SUMOylation site affected LSP1 expression, we measured mRNA levels in HEK293T cells transfected with either His-LSP1 or His-LSP1K270A plasmids. The levels of LSP1 mRNA did not differ between wild-type and mutant LSP1K270A plasmid-transfected cells at 12 h post-transfection (Figure 4A). These results suggest that the reduction in steady-state LSP1 levels due to deSUMOylation is linked to increased protein degradation.

### 2.5. Enhanced Proteasomal Degradation of deSUMOylated LSP1 Due to Increased Ubiquitination

Proteasomal degradation represents a primary pathway for protein degradation in eukaryotic cells. To ascertain whether deSUMOylated LSP1 undergoes proteasomal degradation, HEK293T cells were transfected with His-LSP1, His-LSP1K270A, or co-transfected with His-LSP1 and SENP1, followed by treatment with or without the proteasome inhibitor MG132 (10 μM) for 12 h. Immunoblotting of pulled-down His-tagged proteins using anti-LSP1 rabbit serum demonstrated that, in the absence of MG132, LSP1 degraded significantly more rapidly when SUMOylation was inhibited, either through mutation of the SUMOylation site or by co-expression of SENP1. MG132 treatment rescued both wild-type and mutant LSP1 from proteasomal degradation; however, this recovery was more pronounced in the mutant LSP1^K270A^ and SENP1-overexpressed groups compared to the wild-type LSP1 group (Figure 4B).

In eukaryotic cells, polyubiquitination frequently directs proteins for proteasomal degradation. To examine the involvement of the ubiquitin-proteasome pathway in the accelerated degradation of deSUMOylated LSP1, HEK293T cells were co-transfected with His-LSP1 or His-LSP1K270A and HA-tagged ubiquitin, followed by treatment with or without MG132 (10 μM) for 12 h. Immunoblotting of purified His-tagged proteins with an anti-ubiquitin antibody revealed that in the absence of MG132, ubiquitination of the mutant LSP1 was significantly higher compared to wild-type LSP1 (Figure 4C). Upon MG132 treatment, a markedly increased level of ubiquitination was observed in mutant LSP1 compared to the untreated condition (Figure 4C). In contrast, MG132 treatment led to only a slight increase in ubiquitination of wild-type LSP1. These findings collectively indicate that SUMOylation protects LSP1 from proteasomal degradation, whereas deSUMOylation enhances ubiquitination and accelerates its proteasomal degradation.

### 2.6. SUMOylation Deficiency Impairs Nucleus-to-Extranuclear Translocation of LSP1

**Figure 5:**
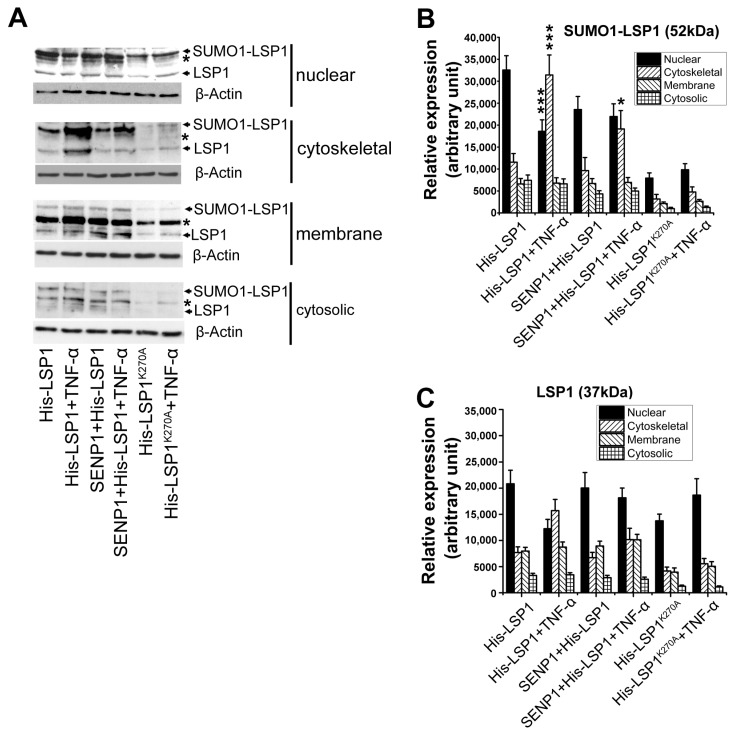
SUMOylation deficiency impairs the nucleus-to-extranuclear translocation of LSP1. (**A**) SVEC4-10EE2 cells were transfected with His-LSP1 and SENP1, His-LSP1, or His-LSP1^K270A^ plasmids. After 12 h, cells were treated with or without 500 ng/mL TNF-α for 4 h. Six million cells from each group were then subjected to subcellular fractionation, and four fractions (in the following order: cytosolic, membrane, nuclear, and cytoskeletal) were collected. Proteins from all four fractions were immunoblotted with anti-LSP1 rabbit serum (relative to β-actin in the cytosolic fraction; *n* = 3). The arrow indicates LSP1 or SUMO1-LSP1 conjugate, and * indicates an unknown signal. (**B**) Densitometric analysis of SUMO1-LSP1 conjugate bands (52 kDa) and (**C**) LSP1 bands (37 kDa) in four subcellular fractions before and after TNF-α treatment. Data are presented as mean ± SD (arbitrary unit; *n* = 3) of three independent experiments. * indicates *p* < 0.05, and *** indicates *p* < 0.001 compared to without TNF-α treatment.

SUMOylation has frequently been associated with the compartmentalization [22,30] and translocation [36,37] of various proteins. In endothelial cells, LSP1 is unevenly distributed across subcellular compartments, with the nucleus as the predominant location [6]. Activation of endothelial cells by TNF-α induces the translocation of some nuclear LSP1 into extranuclear compartments [10]. We investigated whether SUMOylation regulates the compartmentalization and translocation of LSP1. To this end, we overexpressed wild-type LSP1, wild-type LSP1 with SENP1, or mutant LSP1K270A in SVEC4-10EE2 cells, and treated these cells with or without TNF-α. Immunoblotting following subcellular fractionation revealed that LSP1 SUMOylation occurs in all four analyzed subcellular compartments (Figure 5A), with the majority of SUMOylated LSP1 remaining in the nucleus of unstimulated endothelial cells. Notably, mutant LSP1K270A was found almost exclusively in the nucleus, and its translocation from the nucleus to extranuclear compartments upon TNF-α stimulation was minimal (Figure 5A–C). Additionally, the co-expression of SENP1 appeared to inhibit the cytoskeletal translocation of wild-type LSP1. These results suggest that SUMOylation plays a role in the nucleus-to-extranuclear translocation of LSP1.

## 3. Discussion

This study provides compelling evidence that both recombinant and native endothelial LSP1 undergo SUMOylation, a posttranslational modification that plays a critical role in regulating protein function. We demonstrate that this process is mediated by Ubc9, the sole SUMO E2 conjugating enzyme, and reversed by SENP1, a SUMO-specific protease. Through mutational analysis, we identified two lysine residues, K270 and K318, located in the C-terminal region of LSP1, as the primary SUMOylation sites. SUMOylation of LSP1 enhances its stability by protecting it from ubiquitin-mediated proteasomal degradation and facilitates its nuclear export, contributing to its subcellular redistribution.

Although LSP1 is predicted to have a molecular weight of 37 kDa, it consistently appears at ~52 kDa in SDS-PAGE analyses [1,8,38,39]. This shift, observed in both primary endothelial cells and endothelial cell lines, suggests the presence of posttranslational modifications. Using recombinant expression systems and mass spectrometry, we confirmed that the 52 kDa form corresponds to SUMOylated LSP1. Ubc9 overexpression increased, while SENP1 reduced, this modified form, confirming the SUMOylation of LSP1 in endothelial cells. The relative abundance of the SUMOylated fraction of SUMO-modified proteins is typically low, although exceptions have been reported [40]. This variability is likely influenced by factors such as cell lysis conditions and the composition of the lysis buffer [40].

Interestingly, mouse LSP1 lacks the canonical SUMOylation motif (ΨKxD/E); however, recent studies have shown that SUMOylation can occur independently of this consensus sequence. Proteins such as CtBP2, Mdm2, and Daxx, which lack the motif, are still SUMOylated [41,42,43], and in some cases, mutation of the motif does not abolish SUMOylation [44]. Moreover, a non-canonical SUMOylation motif has also been reported [45]. Using SUMOplot™ [22,23,33], we identified K318 in mouse LSP1 as a high-probability SUMOylation site, with conserved sites in human LSP1 at K279 and K327. Site-directed mutagenesis revealed that K270 and K318 are indeed the major SUMOylation sites in murine LSP1, while mutation at K321 had no significant effect, validating the specificity of these modifications.

Functionally, SUMOylation modulates several aspects of protein biology, including stability, subcellular localization, and transcriptional regulation [37,46,47]. Our data show that disruption of SUMOylation—either by mutating key lysines or overexpressing SENP1—leads to a significant reduction in LSP1 steady-state protein levels without affecting mRNA expression, suggesting enhanced degradation rather than decreased synthesis. Cycloheximide chase assays confirmed that SUMOylation-deficient LSP1 has a significantly shorter half-life, and this degradation was rescued by the proteasome inhibitor MG132, indicating a proteasome-dependent mechanism.

Further investigation revealed that SUMOylation prevents LSP1 ubiquitination. While overexpression of ubiquitin led to detectable LSP1 ubiquitination, this was markedly enhanced in SUMOylation-deficient LSP1 and only became evident in the presence of MG132. These findings are consistent with previous reports suggesting that SUMOylation can protect proteins from ubiquitination [16,34,48] by blocking lysine residues [49] or by obstructing recognition by ubiquitin ligases [34,50]. For LSP1, the latter is more plausible, as SUMO and ubiquitin do not share the same lysine sites.

We also explored the role of SUMOylation in the subcellular localization of LSP1. Subcellular fractionation of SVEC4-10EE2 cells showed that wild-type LSP1 is distributed across nuclear and extranuclear compartments and undergoes TNF-α–induced translocation, particularly to the cytoskeleton. This translocation was diminished in cells overexpressing SENP1 or expressing the SUMOylation-deficient LSP1 mutant, indicating that SUMOylation facilitates LSP1 nuclear export. These results align with previous findings that SUMOylation can regulate both nuclear import [51,52] and export [36,53,54] of various proteins.

In conclusion, our study reveals that endothelial LSP1 is subject to SUMO1-mediated posttranslational modification, which enhances its stability by preventing proteasomal degradation and regulates its subcellular trafficking. These findings provide new insights into the regulation of LSP1 and suggest that its SUMOylation may play an important role in endothelial function, including vascular permeability and transendothelial migration of cells, such as immune cells and cancer cells. Future studies will explore the functional consequences of LSP1 SUMOylation in pathological contexts such as inflammation and tumor progression.

## 4. Materials and Methods

### 4.1. Animals

Five- to seven-day-old 129/SvJ mice, sourced from Jackson Laboratories (Bar Harbor, ME, USA), were used in this study with approval from the University Committee on Animal Care and Supply (UCACS) at the University of Saskatchewan.

### 4.2. Plasmids

The HA-ubiquitin plasmid (Addgene plasmid #18712; Addgene, Cambridge, MA, USA) was provided by Dr. Edward Yeh [55]. The plasmids pCMV-SPORT6-LSP1, pCMV-SPORT6-Ubc9, pCMV-SPORT6-SUMO1, and pCMV-SPORT6-SENP1 were purchased from Thermo Fisher Scientific (Burlington, ON, Canada), amplified in E. coli DH5α, and extracted with a Midi Plasmid Kit (QIAGEN, Toronto, ON, Canada). To introduce mutations at potential SUMOylation sites, we performed a one-step mutation PCR (Life Technologies Inc., Burlington, ON, Canada) on the mouse pCMV-SPORT6-LSP1 plasmid. The following primers were designed to replace lysine (K) residues at positions K270, K318, or K321 with alanine (A):

1. Mouse LSP1^K270A^ forward: AGTCAGTCTGCTTCTGCGACACCCTCCTGCCAG;
2. Mouse LSP1^K270A^ reverse: CTGGCAGGAGGGTGTCGCAGAAGCAGACTGACT;
3. Mouse LSP1^K318A^ forward: GCCACTGGACATGGGGCGTACGAGAAAGTACT;
4. Mouse LSP1^K318A^ reverse: AGTACTTTCTCGTACGCCCCATGTCCAGTGGC;
5. Mouse LSP1^K321A^ forward: CATGGGAAGTACGAGGCAGTACTTGTGGATGAGGG;
6. Mouse LSP1^K321A^ reverse: CCCTCATCCACAAGTACTGCCTCGTACTTCCCATG.

The plasmids with the mutation sites were confirmed by sequence analysis.

### 4.3. Cell Culture

Human embryonic kidney 293T (HEK293T; Cat#CRL-3216) cells and the murine microvascular endothelial cell line SVEC4-10EE2 (Cat#CRL-2167) were obtained from the American Type Culture Collection (Manassas, VA, USA). These cells were cultured in Dulbecco’s Modified Eagle’s Medium (Corning, Tewksbury, MA, USA; Cat#10013CV), supplemented with 10% fetal bovine serum (Cytiva Hyclone, Marlborough, MA, USA; Cat#SH30071.03HI), penicillin-streptomycin (100 units/mL and 100 µg/mL, respectively; Gibco, Waltham, MA, USA; Cat#15140122) in a cell culture incubator set at 37 °C and supplied with 5% CO_2_.

### 4.4. Harvest of Murine Primary Endothelial Cells

Primary vascular endothelial cells were isolated from the lungs (LVEC) or hearts (HVEC) of 5–7-day-old pups (129/SvJ) following a previously described protocol [6,56]. Briefly, endothelial cells were isolated using anti-ICAM-2 (CD102) antibody (clone 3C4; BD Pharmingen, Quebec City, QC, Canada; Cat#553326) and cultured on laminin-coated 35 mm Petri dishes. After reaching confluence, the cells were passaged once onto laminin-coated 6-well plates before being used in experiments.

### 4.5. Cycloheximide Chase Assay

The cycloheximide chase assay was performed as previously reported [35], with minor modifications. HEK293T cells were co-transfected with 2 μg of His-LSP1 or His-LSP1K270A and 2 μg of SUMO1. Twelve hours post-transfection, cells were treated with 100 μg/mL cycloheximide (Sigma-Aldrich, Oakville, ON, Canada; Cat#C1988-1G) and then chased at 37 °C for 0 to 12 h. At each time point, cells were washed with cold PBS and lysed in Ni-NTA lysis buffer (50 mM NaH2PO4, 300 mM NaCl, 0.05% Tween, pH 8.0) containing 10 mM imidazole and a protease inhibitor cocktail (Sigma-Aldrich; Cat#P8849) on ice. His-tagged proteins were purified using Ni-NTA slurry, resolved by SDS-PAGE, and immunoblotted with an anti-LSP1 antibody. Bands were quantified using ImageJ software. Relative protein quantities for each time point were analyzed using linear regression, plotted in Origin 9.1 (OriginLab Corporation, Northampton, MA, USA), and half-life values were calculated from the slopes of the fitted curves.

### 4.6. Proteasome Inhibition and In Vitro Ubiquitination Assay

HEK293T cells were co-transfected with His-LSP1 and SUMO1, His-LSP1, SUMO1, and SENP1, or His-LSP1K270A and SUMO1. After 12 h, cells were treated with the proteasome inhibitor MG132 (10 μM; Millipore Canada Ltd., Etobicoke, ON, Canada; Cat#474790) or vehicle for 12 h, lysed, and proteins were immunoblotted with an anti-LSP1 antibody (a generous gift from Dr. J. Jongstra, University of Toronto). For the in vitro ubiquitination assay, HEK293T cells were co-transfected with His-LSP1, SUMO1, and HA-Ubiquitin; His-LSP1, SUMO1, SENP1, and HA-Ubiquitin; or His-LSP1K270A, SUMO1, and HA-Ubiquitin in the presence or absence of MG132 (10 μM; 12 h). Cells were then rinsed with ice-cold PBS and lysed in Ni-NTA lysis buffer containing 10 mM imidazole and a protease inhibitor cocktail (Sigma-Aldrich; Cat#P8849) on ice. His-tagged proteins were purified using Ni-NTA slurry. Proteins were resolved by SDS-PAGE and immunoblotted with an anti-ubiquitin antibody (Sigma-Aldrich; Cat#SAB4503053) [48].

### 4.7. Subcellular Fractionation

Four subcellular fractions were prepared using the Calbiochem ProteoExtract Subcellular Extraction Kit (Millipore Canada; Cat#539790) following the supplier’s protocol [10]. Confluent monolayers of SVEC4-10EE2 cells transfected with His-LSP1 and SUMO1, His-LSP1, SUMO1, and SENP1, or His-LSP1K270A and SUMO1 were subjected to sequential lysis with four different buffers (I–IV), each supplemented with protease inhibitor cocktails and necessary enzymes. The fractions (Fraction I: cytosol; Fraction II: membrane/organelle; Fraction III: nuclear material; Fraction IV: cytoskeleton) were collected and used for immunoblotting.

### 4.8. Nickel-Affinity Pull-Down

Nickel-affinity pull-down under native conditions was performed at 4 °C as previously described [22]. HEK293T cells transfected with various expression plasmids and treatments were lysed in Ni-NTA lysis buffer containing 10 mM imidazole and protease inhibitor cocktail (Sigma-Aldrich; Cat#P8849) on ice. Cell lysates were centrifuged at 10,000× *g* for 10 min to remove debris. His-tagged proteins were pulled down overnight using Ni-NTA agarose slurry (QIAGEN, Toronto, ON, Canada; Cat#30230) and washed five times with lysis buffer containing 10 mM imidazole. Proteins were eluted in a lysis buffer containing 250 mM imidazole.

### 4.9. Immunoblotting

Proteins from pulled-down samples, whole cell lysates, or subcellular fractions were subjected to immunoblotting as previously described [56]. Briefly, protein samples were solubilized in Laemmli sample buffer (Sigma-Aldrich), resolved by 10% SDS-PAGE, and transferred to nitrocellulose membranes. After blocking with 5% non-fat milk, membranes were incubated overnight at 4 °C with primary antibodies against SUMO1 (Life Technologies, Burlington, ON; Cat#MA5-35272), LSP1 (Cat#sc-271137), His (Cat#sc-57598), or β-actin (Cat#sc-130065) (Santa Cruz Biotechnology, Santa Cruz, CA, USA). Following incubation with horseradish peroxidase-conjugated secondary antibodies (Santa Cruz Biotechnology) for 1 h at room temperature, antibody binding was detected using enhanced chemiluminescence detection reagent (GE Healthcare, Baie d’Urfe, QC, Canada). Bands were quantified with ImageJ software (v 1.47).

### 4.10. Mass Spectrometry

After resolving in 10% SDS-PAGE, the proteins were stained with Coomassie Brilliant Blue or Silver Stain. Protein bands of interest from the stained gels were excised and processed using a MassPrep Station (Waters, Milford, MA, USA) as per the manufacturer’s instructions [56]. Gel fragments were destained, reduced, alkylated, digested with trypsin, and extracted overnight at room temperature. The tryptic digest was analyzed by mass spectrometry. For electrospray, quadrupole time-of-flight (Q-TOF) analysis, 1 μL of the solution was used. Liquid chromatography/mass spectrometry (LC/MS) was performed on a CapLC high-performance liquid chromatography system (Waters) coupled to a Q-TOF-2 mass spectrometer (Waters). A mass deviation of 0.2 was allowed with one missed cleavage site. Data were searched against the SwissProt database for Rodentia using the Mascot search engine (www.matrixscience.com).

### 4.11. Real-Time Quantitative PCR

Total RNA was extracted from HEK293T cells transfected with His-LSP1 or His-LSP1K270A using the RNeasy Mini Kit (QIAGEN; Cat#74104). cDNA was generated by reverse transcription PCR with the QuantiTect Reverse Transcription Kit (QIAGEN; Cat#205311). Relative mRNA levels were measured with the QuantiTect SYBR Green PCR Kit (QIAGEN) and predesigned primers for mouse LSP1 (QT01046227; QIAGEN) and human β-actin (QT00095431; QIAGEN). qPCR was performed using an iCycler iQ apparatus (Bio-Rad, Hercules, CA, USA), with human β-actin serving as the housekeeping gene. All PCRs were conducted in triplicate according to the supplier’s protocol [57].

### 4.12. Confocal Microscopy

SVEC4-10EE2 cells were cultured on glass coverslips, co-transfected with GFP-LSP1 and pCMV-SPORT6-SUMO1, and incubated for 24 h. The medium was removed, and cells were washed with pre-warmed PBS before fixation in 4% formaldehyde for 30 min. Cells were then washed with PBS, permeabilized with 0.1% Triton X-100, and blocked with 5% BSA for 30 min. Cells were incubated with mouse anti-SUMO1 monoclonal antibody (Life Technologies) for 2 h at room temperature, washed with PBS, and incubated with Alexa Fluor 532-conjugated goat anti-mouse IgG (Life Technologies) for 1 h in the dark. Coverslips were mounted on glass slides using ProLong Gold anti-fade reagent (Life Technologies; Cat#P36930), sealed with nail polish, and visualized using a laser scanning confocal microscope equipped with LSM700 software (Carl Zeiss Canada Ltd., Toronto, ON, Canada) [57].

### 4.13. Statistical Analysis

Data are presented as the arithmetic mean ± SD. Statistical analyses were performed using Student’s *t*-test or one-way ANOVA with Tukey’s post hoc comparison test. The notation “*n*” refers to the number of different cell batches studied in each group. Values of *p* < 0.05 were considered statistically significant.

## Figures and Tables

**Table 1:** The high probability SUMO1 acceptor sites in mouse and human LSP1 as predicted by SUMOplot.

No.	Species	Position	Group	Score
1	Mouse	K318	VATGH **GKYE** KVLVD	0.67
	Human	K327	VATGH **GKYE** KVLVE	0.67
2	Mouse	K270	QSQSA **SKTP** SCQDI	---
	Human	K279	QAQSA **AKTP** SCKDI	0.69

Sequences like the consensus SUMOylation motif are highlighted in bold, and the potential SUMO acceptor lysine residues are underlined.

## Data Availability

The original contributions presented in this study are included in the article. Further inquiries can be directed to the corresponding author.

## Abbreviations

The abbreviations used are: SUMO, small ubiquitin-like modifier; LSP1, leukocyte-specific protein 1; Ubc9, ubiquitin conjugating enzyme 9; SENP1, SUMO1/sentrin-specific protease 1; SAE, SUMO-activating enzyme; PIAS, protein inhibitor of activated STAT; TNF-α, tumor necrosis factor-α; HEK293T, human embryonic kidney 293T; HVEC, heart vascular endothelial cells; LVEC, lung vascular endothelial cells; ICAM-2, intercellular adhesion molecule-2; Ni-NTA, nickel nitrilo triacetic acid.
